# Therapeutic potential of human embryonic stem cell transplantation in patients with cerebral palsy

**DOI:** 10.1186/s12967-014-0318-7

**Published:** 2014-12-12

**Authors:** Geeta Shroff, Anupama Gupta, Jitender Kumar Barthakur

**Affiliations:** Nutech Mediworld, H-8, Green Park Extension, New Delhi, 110016 India; Pediatrician, Max Hospital, Saket, New Delhi, India; Ministry of Home Affairs, Government of India, New Delhi, India

**Keywords:** Human embryonic stem cells, Cerebral palsy, Transplantation, GMFCS-E & R

## Abstract

**Background:**

The present study evaluated the efficacy and safety of human embryonic stem cell (hESC) therapy in patients with CP.

**Materials and methods:**

This analysis included patients (30 days-18 yr) with documented diagnosis of CP. The study consisted of four treatment phases (T1, T2, T3, T4) separated by gap phases. Efficacy of hESC therapy was evaluated based on Gross Motor Function Classification Scores Expanded and Revised (GMFCS-E & R; 1-good to 5-bad).

**Results:**

Ninety one patients were included and all received hESC therapy in T1, 66 patients returned for T2, 38 patients for T3, and 15 patients for T4. Overall, 30.2% patients achieved GMFCS-E & R score 1 during the study with different number of patients achieving GMFCS score 1 by the end of each treatment phase (T1: 6 [6.6%]; T2: 7 [10.6%]; T3: 11 [28.9%]; and T4: 5 [33.3%]). All patients in up to 2 yr (n = 10), 2-4 yr (n = 10), 4-6 yr (n = 9), and 6-12 yr (n = 8) age groups except one of the 5 patients in the age group of 12-18 yr transitioned from GMFCS-E & R score 5 to lower scores by end of T1. Most patients transitioned to GMFCS-E & R score 2 (n = 34) from higher scores by end of T2. Eleven patients achieved GMFCS-E & R score 1 by end of T3. No serious adverse events were observed.

**Conclusion:**

Use of hESC therapy in patients with CP is effective and safe. hESC therapy has demonstrated significant improvement in GMFCS-E & R scale.

## Introduction

Cerebral palsy (CP), a leading cause of disability among children is caused by damage to the developing brain. According to the Centers of Disease Control (CDC), prevalence of CP around the world ranges from 1.5 to more than 4 per 1,000 live births [1]. In India, the prevalence rate of CP per 100,000 population was estimated to be 282.70 (95% CI 208.43-374.82) in a cross-sectional observation study [1].

Stem cell therapy has the potential to overcome neurological impairments caused by CP. This is achieved by their ability to replace damaged cells of the nervous system. The replacement and transformation potential is higher with embryonic stem cells (ESC) as compared with other types of stem cells [2]. Human embryonic stem cells (hESCs), which are obtained from early pre-implantation stage human fertilized ovum are self renewing cells capable of differentiating into any cell type in the human body. This ability has led to their extensive use in the treatment of several neurodegenerative and neurological disorders [3].

Embryonic stem cells have shown favorable results in animal models. Zhang *et al* found favorable post-implantation histological changes in a rat model 24 hr after injury with survival of the transplanted cells, migration, and differentiation of these cells towards neural cell types [4]. Ma *et al* showed that embryonic-derived stem cells possessed the ability to migrate to the injury site and improve learning ability and memory completely eight months after the injury [5]. Daadi *et al* demonstrated a significant (*p* < 0.05) improvement in locomotor deficits due to CP in animal model after 1 month of stem cell transplantation [6]. Liao *et al* showed that transplantation of cord blood stem cells in animal models showed improvement in neonates with hypoxic ischemic encephalopathy having CP. This improvement resulted from anti-inflammatory effects, release of neurotrophic factors, and by the stimulation of endogenous neurogenesis [7].

However, there is a paucity of data on use of hESCs in patients with CP. The present study evaluated the efficacy of hESC therapy in patients affected with CP using Gross Motor Function Classification System- Expanded and Revised (GMFCS-E & R) in patients aged up to 18 yr. The safety of hESC therapy in the treated patients was assessed in terms of adverse events (AEs) documented at any time during the course of therapy.

## Materials and methods

### Study characteristics

This study was a retrospective analysis of a total cohort of 101 patients with CP who were treated with hESC conducted from 01 October 2007 to 31 July 2013 in New Delhi, India.

### Ethics statement

The study protocol was approved by the Independent Ethics Committee (IEC). The institutional committee for stem cell research and therapy of Nutech Mediworld reported the clinical study to National Apex Body. The study was conducted in accordance to the Declaration of Helsinki [8].

A written informed consent was obtained from the patients/parents/guardians prior to the treatment.

### Study population

Patients aged 30 days to 18 yr and with a documented diagnosis of CP who provided a written informed consent were included. Patients above the age of 18 yr were excluded.

### Cell culture and differentiation

The cells are cultured and maintained as per our proprietary in-house technology (United States Granted Patent No US 8592, 208, 52) in a good manufacturing practice (GMP), good laboratory practice (GLP) and good tissue practice (GTP) certified laboratory. The cell lines are free of animal product and are chromosomally stable. Two directed cell lines, non-neuronal and neuronal were obtained from a single, spare, expendable, pre implantation stage fertilized ovum taken during natural *in vitro* fertilization (IVF) process with due consent. The detailed cell culture and differentiation techniques have been elaborated elsewhere (detailed compositions comprising human embryonic stem cells and their derivatives, methods of use, and methods of preparation is available at http://patentscope.wipo.int/search/en/WO2007141657.

The priming injection of a pharmaceutical composition contained about 750,000 to 80 million hESC and/or their derivatives, resuspended in a volume of about 0.25 - 1.0 ml of sterile normal saline. It was estimated that 1 mL of cells dosage contained 3.5 × 10^6^ hESCs; therefore, 0.25 mL contained 14 × 10^5^ hESCs. The concentration of the cells at the last stage was 2.5-3.5 million cells per mL. These cells were further stored in 1 mL, 2 mL, 5 mL and 10 mL syringes at -20°C for further clinical use. When required the prefilled frozen syringes were thawed by placing the syringes inbetween palms of the hands till they attain the body temperature prior to the transplantation. After this slow thawing process, they were injected into the patient under aseptic conditions. A quality check was performed on the stored cell batches which included integrity, viability and microbial contamination. The cells were characterized and the transplanted cells were octamer-binding transcription factor 4 positive (OCT4 + ve); Stage-specific embryonic antigen 3 (SSEA3) + ve; NANOG + ve; SOX + ve; β actin + ve; β-human chorionic gonadotropin (β–HCG) + ve; alkaline phosphatase + ve; CD 34 + ve; Nestin + ve; GAF + ve; NeuN + ve and transfer gene (TRA) –ve. The characterization was done by fluorescence-activated cell sorting (FACS), polymerase chain reaction (PCR) and immunoflourescence (Nikkon Ellipse E200; BD Acuri, Biorad T 100 Thermal cycle).

### Study design

The study consisted of four treatment phases (T1, T2, T3, T4) with each phase separated by a gap phase. After an established diagnosis of CP through developmental quotient (DQ) and Single Photon Emission Computed Tomography (SPECT) scan, the patients were tested for hypersensitivity reactions with hESC (0.25 mL hESC injected subcutaneously). The SPECT scan was obtained using Millennium MG, GE and was carried out before or within 7 to 10 days of hESC therapy initiation and thereafter at the end of each treatment phase. The patients received an IV injection of hexa methyl propylene aminoxime (HMPAO) into antecubital vein before the SPECT scan. After 15 min to 2 hr of receiving the injection, the patients were placed in a supine position with the orbitomeatal line positioned vertically centered in the field of view.

Following safety evaluations, patients entered the first treatment phase (T1, 8 weeks) where 0.25 mL (<4 million cells) hESCs were administered through intramuscular (IM) route once daily and 1 mL of hESC (<16 million cells) was administered twice every 7 days through intravenous (IV) route. The IM route was used for priming and the IV route was used to facilitate faster transplantation and migration of the hESCs to the affected area. In addition, each patient received a dose of hESCs by caudal route which could be repeated every 5-14 days. hESCs were administered through the caudal route to ensure they reach the spinal fluid and regenerate the spinal cord and allow deep muscles to repair. The patients received eye drops if required 0.25 mL two times a week (administered in patients with visual impairment), nasal spray 0.25 mL two times a week (to facilitate migration of hESCs to the brain through the olfactory nerves), oral drops, ear drops of hESCs were also given to the patients (as per their clinical condition). They were also given deep spinal muscle injections at the back of the neck and base of the spine once a week. The visually impaired children were also given retro bulbar injections. After a gap period of 3-6 months, the patients entered second and third treatment phase (T2 and T3) where they were administered the same dosage regime as T1. Both T2 and T3 lasted for 4 weeks and were separated by a gap phase of 3-6 months. The injections could be increased for an individual, if required.

**Figure 1:**
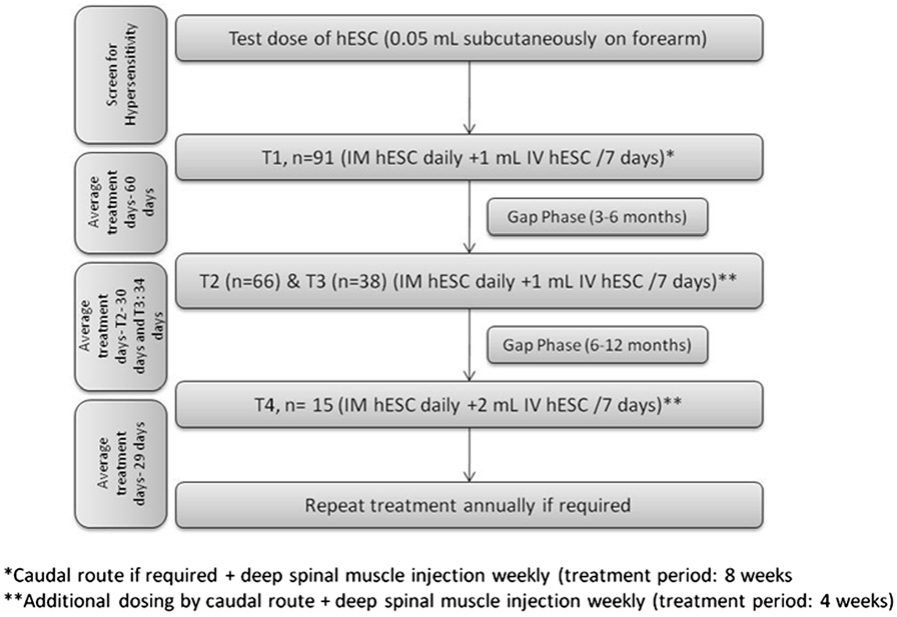
**Study design and disposition of subjects.**

The next treatment phase, T4 was performed after a gap period of 6-12 months. The dosage regimen of T4 was similar to that of T2 however; the IV dose of hESC was increased by 1 mL. The dose of hESCs administered was changed over a period of time as per the patient’s condition and repeated dose of hESCs was administered with extra caution. All the patients received physiotherapy and rehabilitation program in addition to hESC therapy. The study design is illustrated in Figure 1.

In the present study, no control group or placebo group was included. The patient and prognosis observed was considered as a control.

### Variables for analysis

#### Efficacy evaluation

The efficacy of hESC therapy was evaluated based on the GMFCS-E & R scores (1[good] to 5[bad]). These scores were collected at baseline and after completion of each treatment phase.

#### Safety evaluation

The safety of hESC therapy was evaluated by assessing the AEs experienced by patients during the study. Any disabling symptom/sign that a patient suffered after the test dose was given was considered as an AE. The medical staff of Nutech Mediworld carefully examined the patients for any AEs keeping the ones related to hESCs in mind. These AEs included teratomas, and antigen-antibody reactions.

### Data validation

The data for all the patients was validated by Moody International (DOC NO MIC/APR/2010/01), and Quality Austria Central Asia Pvt Ltd (DOC NO Q ACA/OCT/2013/26), accreditation company. These companies were allowed to examine the medical and statistical data present at the institute and were also able to meet the patients.

### Statistical analysis

No formal sample size was calculated for this study. Each case was assessed at admission or soon after admission to determine the pre-therapy status of the case. The GMFCS-E & R scores were calculated for each patient (91 cases). All patients were classified into different age groups *viz* ≤2 yr; 2 to 4 yr; 4 to 6 yr; 6 to 12 yr; and 12 to 18 yr. The analysis was based on frequency or count of the cases placed in different groups. The safety analyses were performed on safety population (patients who took at least one dose of hSEC). All the statistical tests were conducted at 5% level of significance. Statistical analysis was performed using SPSS 19 software (IBM Corporation, Armonk, NY).

## Results

### Study patients

A total of 91 patients were included in the study and all patients were started on intensive dosing. Most patients included in the study were males (71.4%) aged up to 18 yr. All 91 patients were included in T1. Of the 91 patients, 66 patients returned for T2, 38 patients for T3, and 15 patients for T4. The number of patients per treatment phase is illustrated in Figure 1. The total treatment days in T1 were 60 days, in T2 were 30 days, in T3 were 33 days, and in T4 were 29 days.

### Efficacy evaluation

The efficacy of hESC therapy was assessed by evaluating the GMFCS-E & R score at the end of each treatment phase. Each patient who received hESC therapy at the beginning of the study showed a change in GMFCS-E & R score for better between baseline and end of T4. The improvement in GMFCS-E & R score by the end of each treatment period is presented in Table 1.

**Table 1:** **GMFCS-E & R scores of the patients in each treatment phase**

**Treatment phase**	**Age group**	**Number of patients**									
		**GMFCS scores (start of session)**					**GMFCS scores (end of session)**				
		**1**	**2**	**3**	**4**	**5**	**1**	**2**	**3**	**4**	**5**
T1 (n = 91)	≤2 yr (n = 12)	0	0	2	0	10	0	3	5	4	0
	2-4 yr (n = 14)	0	0	1	3	10	0	2	7	5	0
	4-6 yr (n = 21)	0	5	2	5	9	1	8	9	3	0
	6-12 yr (n = 27)	1	3	8	7	8	3	11	10	3	0
	12-18 yr (n = 17)	0	4	2	6	5	2	8	6	0	1
	Overall	1	12	15	21	42	6	32	37	15	1
T2 (n = 66)	≤2 yr (n = 11)	0	3	5	3	0	0	6	3	2	0
	2-4 yr (n = 11)	0	1	6	4	0	1	1	7	2	0
	4-6 yr (n = 12)	0	5	4	3	0	1	7	2	2	0
	6-12 yr (n = 21)	1	8	10	2	0	3	13	5	0	0
	12-18 yr (n = 11)	0	5	5	1	0	2	7	1	1	0
	Overall	1	22	30	13	0	7	34	18	7	0
T3 (n = 38)	≤2 yr (n = 6)	0	2	2	2	0	0	3	3	0	0
	2-4 yr (n = 10)	0	3	5	2	0	2	5	2	1	0
	4-6 yr (n = 7)	0	4	1	2	0	2	3	1	1	0
	6-12 yr (n = 9)	1	5	3	0	0	3	6	0	0	0
	12-18 yr (n = 6)	2	3	0	1	0	4	1	0	1	0
	Overall	3	17	11	7	0	11	18	6	3	0
T4 (n = 15)	≤2 yr (n = 5)	0	4	1	0	0	2	3	0	0	0
	2-4 yr (n = 4)	0	2	2	0	0	2	1	1	0	0
	4-6 yr (n = 2)	0	0	2	0	0	0	1	1	0	0
	6-12 yr (n = 1)	0	1	0	0	0	0	1	0	0	0
	12-18 yr (n = 3)	1	1	1	0	0	1	1	1	0	0
	Overall	1	8	6	0	0	5	7	3	0	0

Overall, of the 91 patients who received hESC therapy in T1, 6 patients (6.6%; 4-6 yr- 1 patient; 6-12 yr- 3 patients; 12-18 yr- 2 patients) were at GMFCS-E & R score of 1 by the end of T1. Forty-two patients had a GMFCS-E & R score of 5 at the beginning of T1; of which 41 patients transitioned to lower scores by the end of T1. By the end of T1, 37 (up to 2 yr- 5 patients; 2-4 yr- 7 patients; 4-6 yr- 9 patients; 6-12 yr- 10 patients; 12-18 yr- 6 patients) transitioned to GMFCS-E & R score 3 and 32 (up to 2 yr- 3 patients; 2-4 yr- 2 patients; 4-6 yr- 8 patients; 6-12 yr- 11 patients; 12-18 yr- 8 patients) to GMFCS-E & R score 2. All patients in the age group of up to 2 yr (10 patients), 2-4 yr (10 patients), 4-6 yr (9 patients), and 6-12 yr (8 patients) transitioned from GMFCS-E & R score 5 to lower scores by the end of T1. Four of the 5 patients with a GMFCS-E & R score 5 in the age group of 12-18 yr transitioned to lower scores by the end of T1 (Table 1).

Overall, 66 patients received hESC therapy in T2, 7 (10.6%; 2-4 yr and 4-6 yr- 1 patient each; 6-12 yr- 3 patients; and 12-18 yr- 2 patients) were at GMFCS-E & R score of 1 by the end of T2. Of the 30 patients (up to 2 yr- 5 patients, 2-4 yr- 6 patients, 4-6 yr- 4 patients, 6-12 yr- 10 patients, and 12-18 yr- 5 patients) with a GMFCS-E & R score of 3 at the beginning of T2, 12 patients transitioned to lower GMFCS-E &R scores by the end of T2. By the end of T2, most patients transitioned to GMFCS-E & R score 2 (34 patients) (Table 1).

Of the 38 patients who received hESC therapy inT3, 11(28.9%; 2-4 yr- 2 patients; 4-6 yr- 2 patients; 6-12 yr- 3 patients; 12-18 yr- 4 patients) were at GMFCS-E & R score of 1 by the end of T3. Majority of the patients who scored 4 on GMFCS-E & R scale (up to 2 yr-2 patients, 2-4 yr- 2 patients; and 4-6 yr- 2 patients; 12-18 yr- 1 patient) at the beginning of T3; transitioned to lower scores (Table 1).

In the last treatment phase T4, hESC therapy was received by 15 patients and 5 (33.3%; up to 2 yr- 2 patients; 2-4 yr- 2 patients; 12-18 yr- 1 patient) were at GMFCS-E & R score of 1 by the end of T4. One of the 4 patients with a GMFCS-E & R score 2 in the age group of up to 2 yr transitioned to a lower GMFCS-E & R score by the end of T4. Three of 6 patients (up to 2 yr- 1 patient; 2-4 yr- 2 patients; 4-6 yr- 2 patients; 12-18 yr- 1 patient) transitioned to lower GMFCS-E & R scores by the end of T4 (Table 1).

Change in the score (worse, no change, and better) was assessed to evaluate the effect of hESC therapy. All patients of ≤2 yr and 12-18 yr age group showed an improvement in score by at least one score by the end of T1. Higher proportion of patients from the age groups of 2-4 yr (92.9%), 4-6 yr (90.5%), and 6-12 yr (92.3%) showed an improvement in score by the end of T1 (Table 2). In addition, 100% female patients (26/26) and 92.3% of male patients (60/65) showed an improvement in GMFCS-E & R score by at least one score by the end of T4.

**Table 2:** **Number of cases scoring differently by at least one score from baseline to the end of treatment phase 1 (T1)**

**Age group**	**Affected cases**	**Alteration in the score at least by 1 score [n (%)]**		
		**Worse**	**No change**	**Better**
Up to 2 yr	12	0 (0)	0 (0)	12 (100)
2 to 4 yr	14	0 (0)	1 (7.1)	13 (92.9)
4 to 6 yr	21	0 (0)	2 (9.5)	19 (90.5)
6 to 12 yr	27	0 (0)	2 (7.4)	25 (92.6)
12 to 18 yr	17	0 (0)	0 (0)	17 (100)

In addition to GMFCS-E & R scores, we also analyzed the cognitive skills of the patients using additional parameters. The cognitive skills of all the patients was assessed by the end of T4 using 11 parameters (problem solving, following commands, smiling, recognition/awareness, eye contact, aggression, speech, feeding, toilet training, daily living skills, and defense mechanism). This assessment was done to evaluate the improvement in cognition and functional recovery of the patients.

**Figure 2:**
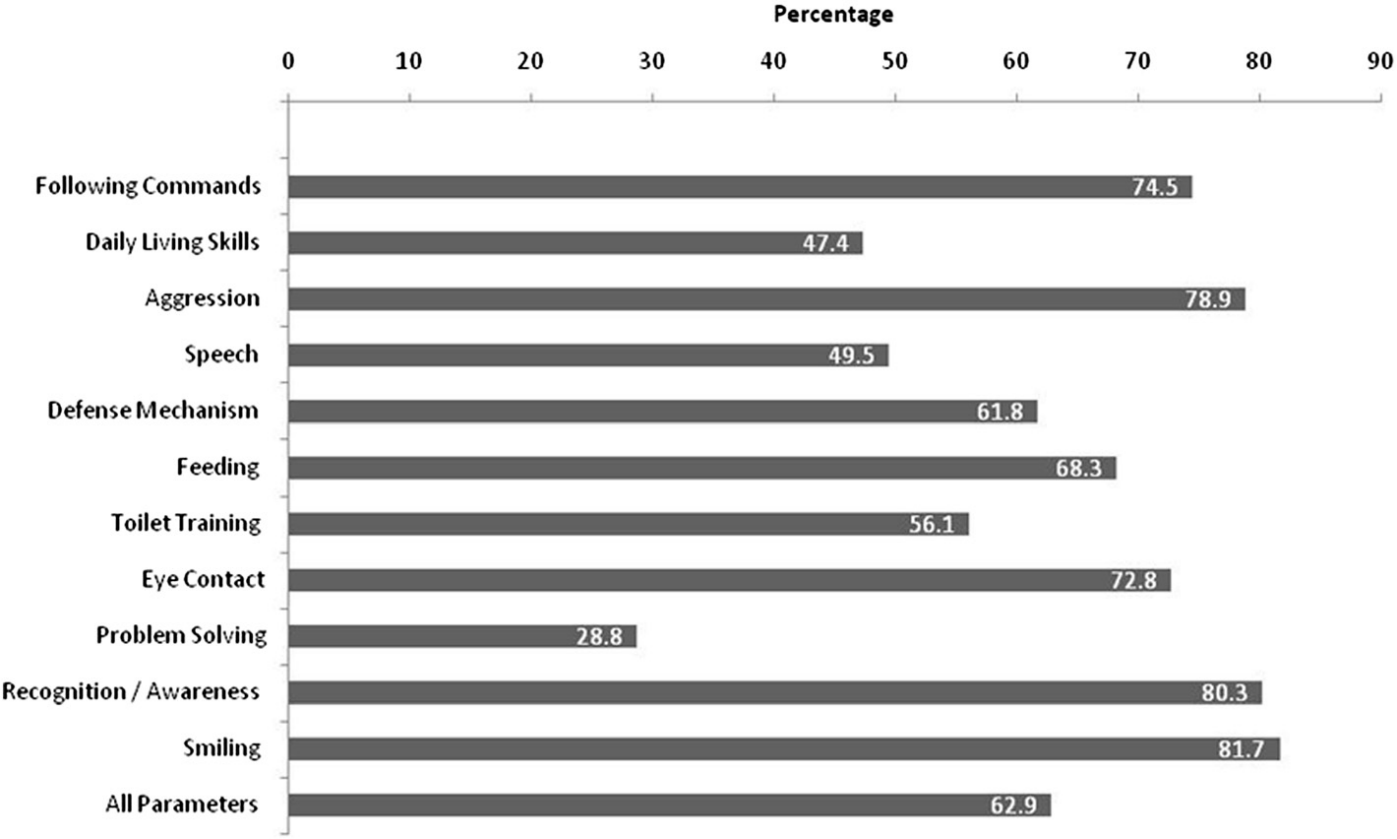
**Cognitive skills by the end of the therapy.**

Overall, 69% of the patients showed an improvement in cognitive skills (Figure 2). Improvements in parameters which were documented using an in house developed scoring system such as smiling (81.7%), recognition/awareness (80.3%), aggression (78.9%), and following commands (74.5%) were higher as compared with other parameters.

Eight patients had hearing impairment of which five were either totally deaf or heard sounds close to the ear. All eight patients showed improvement by the end of T4. A total of 59 patients had seizures before receiving treatment, of which 45 had seizures multiple times a day to 1-2 times every other day. All patients were seizure free however; one patient continued to have seizures every other day by the end of T4.

Overall, 29 patients scored 1, 50 patients scored 2, 12 patients scored 3, and none scored 4 and 5 on the GMFCS-E & R scale by the end of T4. A significant association (*p <* 0.05) between the severity of cases and whether or not they showed improvement in GMFCS-E & R score was observed in the present study. All the patients had received physiotherapy prior to hESC therapy and had shown no improvement.

**Figure 3:**
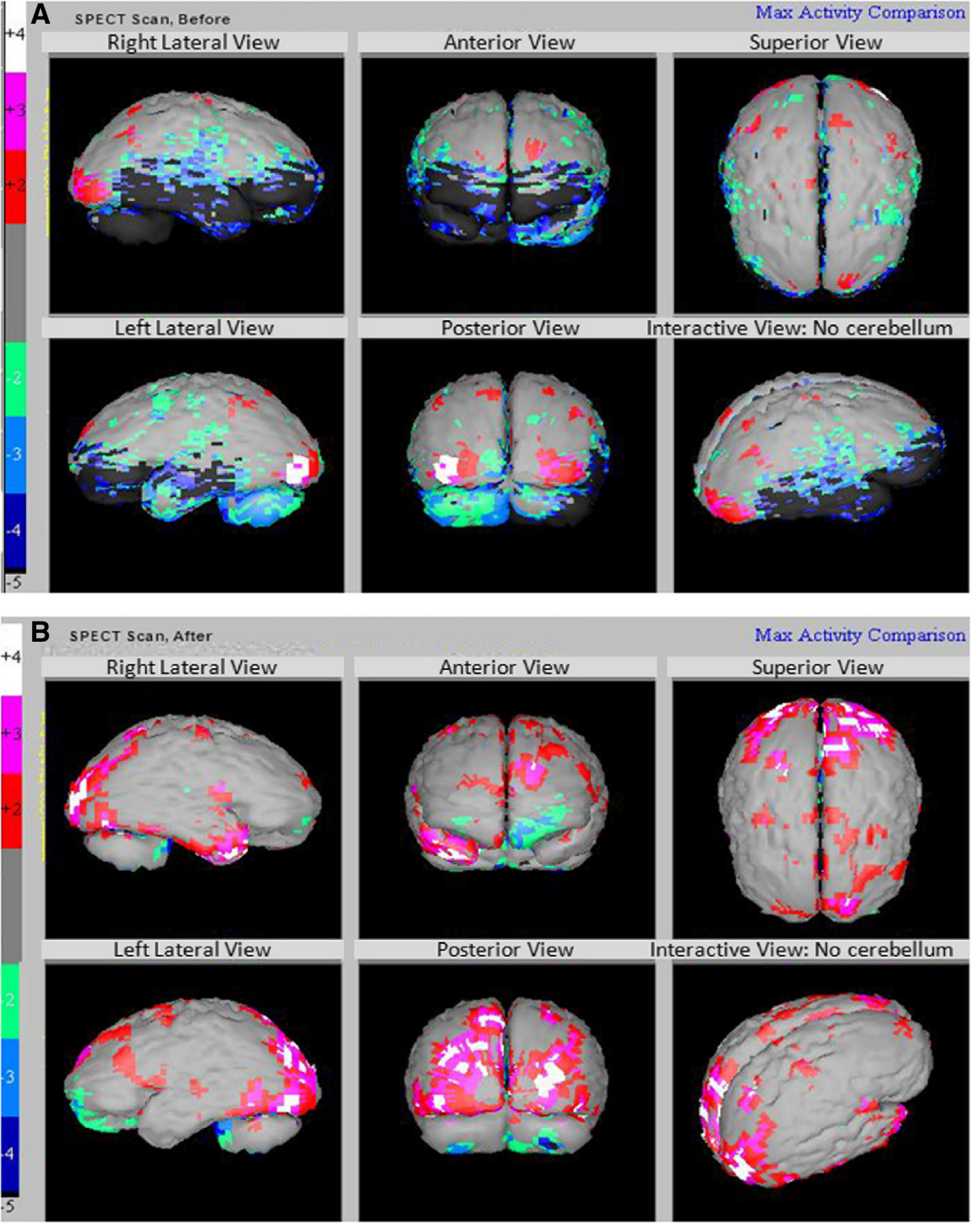
**SPECT scan of a cerebral palsy patient (grey - normal; red, pink and white - above normal; green, light/dark blue, black - hypoperfused). A)** Showing hypoperfused Regions before Receiving hESC Therapy. **B)** Improved Perfusion (reduced black areas) after Receiving hESC Therapy.

We were able to do SPECT scan in all our patients before and after the therapy. The SPECT scan of the patients demonstrated improved perfusion after receiving hESC therapy. The SPECT scan report of one of our patient is illustrated in Figure 3A and B.

### Safety evaluation

The commonly experienced AEs included body pain, diarrhea, chest congestion, throat infection, fever, itching, and swelling. A total of 9 patients (9/91; 9.9%) experienced AEs during T1 (swelling-1, itching-1, fever-4, throat infection-1, and chest congestion-2), 2 patients (2/66; 3%) experienced AEs during T2 (diarrhea-1, and body pain-1), 1 patient (1/38; 2.6%) experienced AEs during T3 (diarrhea-1) and no AE were reported in T4. No serious AEs were reported during the study. The AEs observed during the study are presented in Table 3.

**Table 3:** **Adverse events experienced by patients during the study period**

**Treatment phase**	**Adverse event**	**No. of patients**
T1	Swelling	1
	Itching	1
	Fever	4
	Throat infection	1
	Chest Congestion	2
	Total	9
T2	Diarrhea	1
	Body Pain	1
	Total	2
T3	Diarrhea	1
	Total	1
T4	Nil	-

## Discussion

Cerebral palsy, a neurological disorder that affects children leads to compromised mental health and motor abilities. To our knowledge, this is the first study to assess efficacy and safety of hESC therapy in patients with CP. The use of hESCs has not been clinically viable in the past due to difficulty in harvesting these cells in a xeno free environment. However; we used a patented in-house (Patent-WO 2007/141657A PCT/1B 2007 Published 13 Dec 2007) methodology for the isolation of hESC to explore their potential in the treatment of CP.

All the patients had come to our facility after not benefitting from the physiotherapy and other traditional therapies. At our facility, the patients were given hESC therapy along with physiotherapy. The results of the present study with hESC are promising and showed improvement in considerable number of patients assessed.Bjorgaas *et al* showed that children with CP are often prone to hyperactivity, conduct problems, peer problems and show a prosocial behavior [9]. Children with CP also show abnormal posture and movement problems. Although motor function and posture problems are common among children with CP, emotional quality of life (QoL) and social well being is affected in these children to a significant extent [10]. According to a questionnaire based study, the most important aspects of treating patients with CP included improvement in function, mobility, activity, participation, and QoL [11].

Although several therapies are available for the treatment of patients with CP, none have shown satisfactory outcomes. Rehabilitation [12], physical therapy [13], nutritional support for malnourished CP patients [14], and oral motor therapy for patients with difficulty in swallowing, drooling, chewing problems [15], and the use of botulinum toxin A [16] are few therapies available for the treatment of patients with CP. However; the use of botulinum toxin A in patients with CP is still conflicting. In our study, the rehabilitation programme was provided only during the therapy. hESCs transplantation was the mainstay of the treatment. Though all the patients had undergone rehabilitation programme prior to treatment with hESCs, but they were not benefited.

Neonatal hypoxic-ischemic brain injury is one of the major causes of CP. Patients with CP experience loss of oligodendrocytes and myelin. The neural stem cells and glial progenitor cells (GPCs) have the ability to differentiate to form oligodendrocytes, and astrocytes resulting in improved outcomes in the treatment of CP [17]. Stem cells replace the damaged neurons through a chain of reactions involving both exogenous and endogenous neurons, glial cells, and endothelial cells. These cells act synergistically to repair brain damage which may possibly have caused CP [18].

Studies related to the use of hESC therapy in patients with CP are few however; the use of cord stem cells in CP patients has been evaluated for efficacy in studies from the past. Transplantation of cord stem cells in a 2.5 year old boy with CP demonstrated significant improvement in motor function at week 4 of therapy, normal EEG and partial recovery from vision loss at week 7 of therapy [19]. Another study examining the efficacy of stem cells in a 6 year old CP patient showed significant improvement in motor function within 6 months of therapy and also showed cognitive, speech, and sensory improvements [20]. Chen *et al* demonstrated improvements in gross motor function measurement scores, and language quotients in patients with CP after 3 months (*p*-0.011) and 6 months (*p*-0.001) of therapy with neural stem cells [21].

The GMFCS-E & R scores include the age band of up to 18 yr [22]. As per this revised scale, we included patients aged up to 18 yr for the evaluation of efficacy of hESC therapy with GMFCS-E & R scores. The results demonstrated that the use of hESC therapy in patients with CP was beneficial. Overall, 30.2% patients achieved a score of 1 on GMFCS- E& R scale by the end of T4 of 338 days. A total of 86 patients (94.5%) showed an improvement in GMFCS-E & R score by the end of T4 with hESC. The GMFCS-E &R scale does not consider cognitive improvement as a parameter [23]; however, cognition is an important concern for patients with CP. In the present study we evaluated the improvements in cognitive skills among patients who had received hESC therapy. Overall, cognitive skills of the patients also showed a significant improvement in 69% of the patients included in the study. Of these, highest improvement was observed in parameters including smiling (81.7%), and recognition/awareness (80.3%) In addition to these parameters, hESC therapy showed an improved vision in patients (will be presented in a separate paper). Previous studies have shown that stem cell therapy may be beneficial in the treatment of visually impaired CP patients [24]. The first clinical trial of autologous umbilical cord blood reinfusion in children aged 12 mon to 6 yr with CP is ongoing at the Duke Univeristy. In this randomized, double blind study the participants were/will be treated with autologous cord blood reinfusion. The investigators have hypothesized infusion of autologous umbilical cord blood might facilitate neural cell repair resulting in improved function. The study will be completed in 2016 [25].

Various studies have demonstrated the migration and differentiation of hESCs derived progenitor cells at the site of brain injury [5,26], raising the hope that hESC therapy may eventually be developed to treat disorders related to the central nervous system [27]. hESCs derived precursors have ability to travel along the olfactory system and contribute to neurogenesis [28]. A pre clinical study by Ma *et al* showed that ESCs-derived cells transplanted on hypoxic-ischemic encephalopathy (HIE) mouse model migrated into the injury site of the brain and expressed neural stem cell differentiation markers like Nestin and MAP-2 [5]. In another study by Liu *et al*, the migration of transplanted embryonic mesenchymal stem cells (eMSCs) derived from hESCs in an ischemic rat were tracked by staining the brain sections with GFP antibody. The GFP-positive cells were observed in the infarcted region, penumbra and striatum after two days of cells injection. These cells expressed several neuronal markers like MAP-2 and β-tubulin 3, indicating the neuronal differentiation of transplanted eMSCs [29]. In a study by Yang *et al*, hESC-derived neurons and their progenitors survived transplantation for 5 months in the brain of an experimental rat model of Parkinson disease and contributed to the recovery of locomotor function [26].

In our study, hESCs could have migrated to the hypoperfused areas of the brain after transplantation and “home” in the affected regions. As observed in previous studies, the process of homing is regulated by the communication of the SCs with chemokines, cytokines, and growth factors released from the site of injury [30,31].

A previously conducted study demonstrated that intranasal application of MSCs resulted in the migration of these cells to the olfactory bulb, cortex, hippocampus, striatum, cerebellum, brainstem, and spinal cord of rats with Parkinson’s disease. This study showed that 24% of the cells survived for 4.5 months in the brain of the rats resulting in substantial improvement of motor function, of the forepaw 40-110 days after administration of MSCs, and decreased concentrations of inflammatory cytokines in the rats [32]. Various studies have also reported migration and differentiation of hESCs after intraocular injection [33,34]. The size of the hESCs used in our study is less than 1 μm facilitating them to easily permeate through the parenchyma *via* blood brain barrier. The route of administration determines the ability of the hESCs to reach the affected areas of the brain. These cells migrated to the affected area and showed an improvement in perfusion that is evident from the extent of improvement in the patients reflected in the SPECT scan. The changes in SPECT scan and GMFCS-E & R scale may be exclusively due to hESC therapy. Further, the transplanted hESCs could have remained viable in the body of the patients. Recently, researchers from Thailand have demonstrated that ESCs derived from 18 year old frozen embryos have ability to differentiate into multiple cell types similar to those derived from fresh embryos [35].

In the present study no immunosuppresants were used. The transplantation of hESCs showed no immune response in the patients. This may be comparable to surrogacy wherein a complete genetic stranger is accepted in the host, grows, and is delivered by the host. hESC therapy also improved the overall well being of the patients who participated in the study. Most patients who were unable to perform daily activities as a result of CP were able to perform them with lesser effort.

## Conclusion

The use of hESC therapy in patients with CP is effective and safe. hESC therapy has demonstrated significant improvement in GMFCS-E & R scale and most patients transitioned to a better score after completing all the four treatment phases. The use of hESC therapy showed improvement in cognitive skills CP patients. However; scarcity of data related to hESC therapy and its use in the treatment of CP demands more research in this area.

## Abbreviations

CP: Cerebral palsy
CDC: Centers of Disease Control
ESC: Embryonic stem cells
hESCs: Human embryonic stem cells
GMFCS-E & R: Gross Motor Function Classification Scores Expanded and Revised
AEs: Adverse events
IEC: Independent Ethics Committee
GMP: Good manufacturing practice
GLP: Good laboratory practice
GTP: Good tissue practice
IVF: In vitro fertilization
OCT4 + ve: Octamer-binding transcription factor 4 positive
SSEA3: Stage-specific embryonic antigen 3
β –HCG: β-human chorionic gonadotropin
TRA: Transfer gene
FACS: Fluorescence-activated cell sorting
PCR: Polymerase chain reaction
DQ: Developmental quotient
SPECT: Single Photon Emission Computed Tomography
HMPAO: Hexa methyl propylene aminoxime
IM: Intramuscular
IV: Intravenous
QoL: Quality of life
GPCs: Glial progenitor cells
