# Expression of Concern to: Therapeutic potential of human embryonic stem cell transplantation in patients with cerebral palsy

**DOI:** 10.1186/s12967-017-1292-7

**Published:** 2017-09-18

**Authors:** Geeta Shroff, Anupama Gupta, Jitender Kumar Barthakur

**Affiliations:** 1 Nutech Mediworld, H‑8, Green Park Extension, New Delhi, 110016 India; 2 Max Hospital, Saket, New Delhi, India; 3 Ministry of Home Affairs, Government of India, New Delhi, India

## Expression of Concern: J Transl Med (2014) 12:318 DOI 10.1186/s12967-014-0318-7

The Editor-in-Chief of the *Journal of Translational Medicine* is issuing an editorial expression of concern to alert readers that concerns have been raised regarding the ethics of this study [1] and the potential association of the risk of teratoma formation with the transplantation of embryonic stem cells. Appropriate editorial action will be taken once this has been fully investigated. The authors disagree with this notice.
